# Textile residue-based mycelium biocomposites from *Pleurotus ostreatus*

**DOI:** 10.1080/21501203.2023.2278308

**Published:** 2023-11-15

**Authors:** Rahul Saini, Guneet Kaur, Satinder Kaur Brar

**Affiliations:** a Department of Civil Engineering, Lassonde School of Engineering, York University, Toronto, Ontario, Canada; b School of Engineering, University of Guelph, Guelph, Ontario, Canada

**Keywords:** Cotton, mycelium-based biocomposite, *Pleurotus ostreatus*, polyester, textile waste

## Abstract

The research on mycelium-based biocomposites is increasing exponentially, due to their ability to be produced from renewable and sustainable substrates. In this sense, the present investigation explores the ability of *Pleurotus ostreatus* to grow on textile residues and form mycelium-based biocomposites. The mycelium was able to grow on four types of textile residues including white and coloured cotton and polyester mixtures and acted as a binder between the textile fibres. The growth of fungal mycelium was assessed using Fourier transform infrared spectroscopy to detect the presence of amides and polysaccharides arising from fungal mycelium and scanning electron microscopy, dry weight and water activity. The compressive strength of textile residue-based biocomposite was also measured and it was found to be between 100 and 270 kPa. Overall, a lightweight biocomposite was obtained which could be a potential alternative for polystyrene-based products. These findings show the ability of the fungus to thrive on polyester plastic in textiles and provide an alternative for converting this plastic material into bio-based materials. Additionally, by varying the mycelium growth, the plasticiser and stiffness properties of the resultant biocomposite can be changed. This research paves the way for the efficient conversion of textile waste into biocomposites as alternatives for plastic packaging products.

## 1. Introduction

Over the past decade, plastics and microplastics contamination in the surrounding environment as well as in humans has increased. Hence, potential solutions are being searched to increase the degradation rate of plastics or replace them with renewable and green biodegradable products (Miri et al. 2022). For instance, microbial systems such as fungi are being explored to convert renewable substrates into environment-friendly products. Recently, research on mycelium-based biocomposites has been increasing steadily due to their potential to replace plastic-based packaging products. Mycelium-based biocomposites generally comprise fungal vegetative parts grown on waste residues (Rafiee et al. 2021). The fungus consumes the carbohydrates to produce hyphae, and this cultivation depends on the temperature, moisture and pH of the media. The commonly known fungal species are white-rot basidiomycetes, capable of degrading polymeric carbohydrates such as cellulose and hemicellulose into monomeric forms and using them for their growth (González et al. 2021). For instance, *Pleurotus ostreatus* is an edible white-rot fungus known to degrade cellulose, hemicellulose and lignin, hence reinforcing its potential to thrive on wastes such as wood, textile, and agricultural residues (Huang et al. 2020; Yang et al. 2021).

Biocomposites produced under different conditions could replace the plastics from water bottles, car bonnets or various other appliances (Răut et al. 2021). Biocomposites can be produced using different residues, such as agricultural, domestic or industrial wastes, usually discarded, burned or underutilised (Shanmugam et al. 2021). Out of these wastes, the textile industry could be a potential option for biocomposite production. In general, the textiles industry encompasses daily usage in the fashion and healthcare sectors. Textile fibres are broadly classified as man-made and natural categories. It has been estimated that industries produce more than 100 million metric tons of textile fibre per year, which will increase by 1.5 times by the end of 2030, eventually increasing waste production (Khandaker et al. 2022). However, unlike lignocellulosic biomass, which has been mostly explored for biocomposite production (Tacer-Caba et al. 2020; González et al. 2021; Santos et al. 2021), implementation of textile residues has been hindered by the presence of polyester i.e. plastic compounds, such as polyethylene terephthalate (PET) in such wastes. Therefore, the valorisation of synthetic textiles such as the cotton-polyester blends has generally been considered very difficult, thereby contributing further to the problem of plastic pollution. For instance, Wang et al. (2018) observed a reduced cellulase activity of three fungal strains, *Aspergillus niger, Trichoderma reesei*, and *Trichoderma longibrachiatum* on 100% PET substrates while a higher uninhibited cellulase activity was reported upon use of cotton-based textile wastes. Moreover, most of the studies on textile wastes have been based on the release of monomeric sugars from the cellulose component of textile wastes by biotechnological methods, recycling of the polyester compounds into monomers such as terephthalic acids, or their conversion into products using thermochemical methods (Chopra et al. 2023). For instance, Çay et al. (2020) converted the textile waste (cotton/polyester blend) into biochar and used it to coat cotton cloths which led to increased drying rate, thermal comfort and odour masking. Similarly, textile waste has been studied as reinforcement material for wall sheets (Echeverria et al. 2019), packaging film production (Zhong et al. 2021) and adsorbent production for removal of pharmaceutical residues such as tetracycline and paracetamol from aqueous solution (Akkouche et al. 2021). However, to the best of the authors’ knowledge, no studies are available on textile-based biocomposite production; thus, it is necessary to explore the ability of fungus to grow not only on cotton-based textiles but also on polyester textile residues to produce mycelium-based biocomposites.

In this sense, the present study involves the ability of *Pleurotus ostreatus* to grow and form biocomposites using textile residues. To this end, the study explores four types of textile residues including cotton (white and colour) and polyester (white and colour) textiles. Biocomposite properties were analysed using dry weight, Fourier transform infrared spectroscopy (FTIR), water activity, stress-strain analysis and scanning electron microscope (SEM). The current investigation addresses the potential future of textile-based biocomposites and provides the baseline to further valorise the textile industry wastes while endorsing a sustainable and green environment.

## 2. Materials and methods

### 2.1. Substrate and culture maintenance

*Pleurotus ostreatus* was procured from NRRL culture collection, USA. The fungus was maintained and grown on Potato dextrose agar (PDA) at 30 °C until the Petri plate was completely covered with fungal mycelium and was then stored at 4 °C.

### 2.2. Biocomposite production conditions

Four types of textiles were used in the present study including cotton (white and coloured) and polyester (white and coloured). These were collected from the Salvation Army, Canada. Textile was dried and shredded and three different weights were used i.e. 1 g, 3 g, and 5 g. The moisture content was maintained at 70% using a solution containing 5 g/L of glucose as an additional carbon source and 0.5 g/L ammonium sulphate as a nitrogen source. Textiles were placed in the moulds and sterilised using an autoclave at 121 °C, 15 psi for 20 min and cooled.

After cooling, the textile was inoculated using freshly grown *P. ostreatus* (10% *w/v*) and incubated at 27 °C for 4 weeks, while uninoculated textiles were used as control. The moulds were turned upside-down every third day. In addition, the moisture content was checked everyday using temperature and humidity metre (ERAY, China) and maintained at 75% using autoclaved distilled water. The experiment was performed in duplicates.

### 2.3. Characterization of textile residue-based biocomposites

#### 2.3.1. Dry biomass

The fungal-grown textile was kept at 105 ± 1 °C for 24 h to deactivate the fungus. The difference between before and after-grown textiles was reported in grams.

#### 2.3.2. Water activity

The water activity was calculated based on the relative humidity of samples as described by Kong and Singh (2011). The relative humidity (%) was analysed using temperature and humidity metre (ERAY, China). Following this, the water activity was calculated using the following equation: Water activity = Relative humidity (%)/100.

#### 2.3.3. Fourier transform infrared (FTIR) analysis

FTIR analysis was performed using (VERTEX 70 v, Bruker) in Attenuated Total Reflectance mode at the range of 4,000–400 cm^−1^, 64 scans per spectrum at a resolution of 4 cm^−1^.

#### 2.3.4. Scanning electron microscope

SEM was carried out using Advanced light and an electron microscope. The mycelium biocomposite was cut into 30 mm width and length and 10 mm height and imaged using electrons at an accelerating rate of 30 kV.

#### 2.3.5. Stress and strain analysis

Stress and strain analysis was performed using Trios V4.1 hybrid rheometers-tension fixture at strain from 0–100%, 1 mm/sec tensile speed for 120s. Compressive strength was calculated using a stress-strain curve and force-time profile. All samples were conditioned at 50% relative humidity for three days before the analysis (Tacer-Caba et al. 2020).

### 2.4. Statistical analysis

The fungal biomass growth was evaluated using analysis of variance (ANOVA) using OriginPro®2022 software. The significance was performed at a 95% confidence level using the Fischer test.

## 3. Results

### 3.1. Fungal growth on textile residue

**Figure 1.**
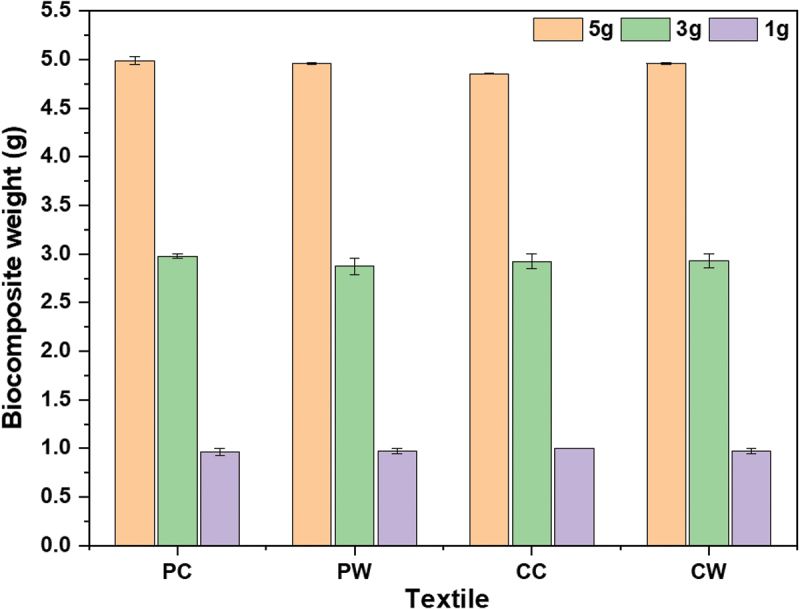
Dry biomass of biocomposite grown with *Pleurotus ostreatus*. PC: Polyester coloured; PW: Polyester white; CC: Cotton coloured; CW: Cotton white.

**Figure 2.**
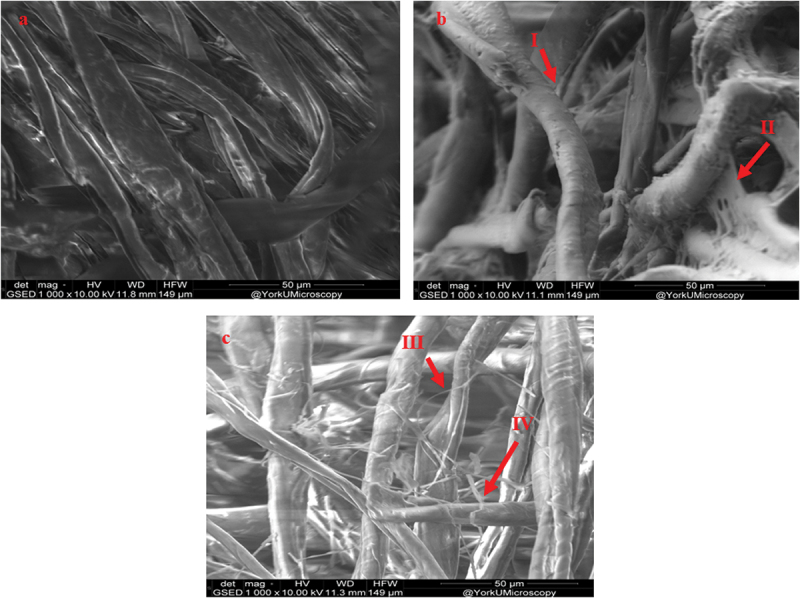
Fungal growth on textile waste and biocomposite development. (a) Textile before inoculation. (b) Textile grown with *Pleurotus ostreatus* (I and II shows fungal growth and crosslinking). (c) Dried biocomposite at 105 °C (III and IV demonstrate the dried and separated hyphae).

Figure 1 illustrates the weight of dry biocomposite grown with the *P. ostreatus*. Moreover, no significant difference in biocomposite weight was observed in the mycelium grown on 1–5 g textile residues (*P* > 0.5). Interestingly, loss of mass was observed in all the samples. After the fungal growth, a decrease of 1%–5% in total dry biomass was observed. Additionally, in the control textile, no mass loss was observed. Moreover, fungal growth and mycelium presence in samples, have been confirmed by visual inspection, water activity, FTIR, and SEM analysis. A decrease in biocomposite weight could be due to the fungal deactivation process, which was performed at 105 °C for 24 h. The mass loss could be due to the water evaporation in samples and mycelium internal moisture loss resulting in the formation of internal voids. Figure 2a, 2b illustrate the textile before inoculation and after fungal growth, while Figure 2c displays the reduction of diameter of mycelium and the formation of internal voids which can be attributed to the evaporation of water present in the fungal cell wall. Moreover, the shape of the biocomposite seemed to be constantly pointing out the fact that volume loss was not more significant than mass loss. The textile mass loss could have happened in the following stages. Firstly, the growth of fungi by degrading the substrate. Secondly, the replacement of textile biomass by lighter weight mycelium. Lastly, there is a loss of mycelium’s internal moisture and sample water during the drying process. Similar results were reported by Santos et al. (2021), where the author studied the effect of heat treatment on *Pycnoporus sanguineus* grown on coconut powder supplemented with 30% wheat bran. The author reported 24.17%, 34.57%, and 42.91% mass loss at 50, 60, and 70 °C for 24 h. In general, moisture retention can cause sample contamination by other microorganisms. Hence, it is really important to remove the moisture content and to deactivate the fungus in the resultant biocomposite. Alternatively, to retain the sampling flexibility and enhance the mechanical properties, the biocomposite can be coated with waterproof material to prevent the loss of fungal internal moisture and avoid direct contact with the external environment.

### 3.2. Morphological and chemical characteristics of textile residue-based biocomposite

**Figure 3.**
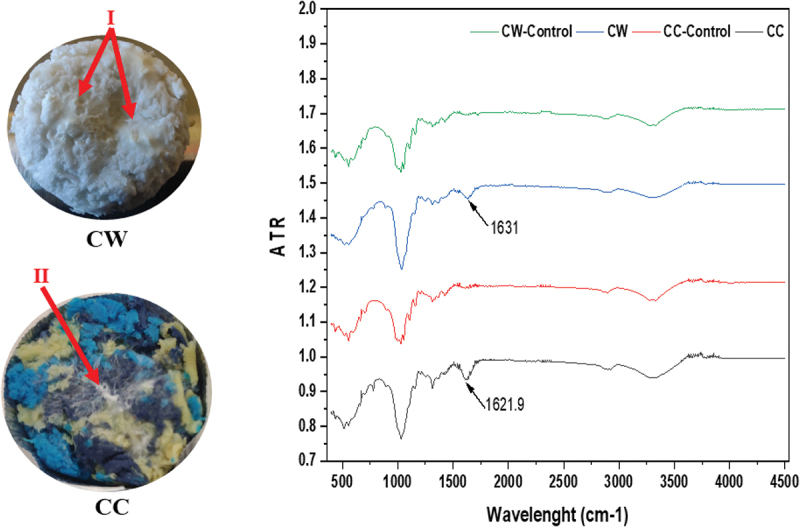
*Pleurotus ostreatus* grown textile (I and II) and the FTIR data. CC: Cotton coloured; CW: Cotton white.

The textile inoculated with *Pleurotus ostreatus* was visually inspected and was flipped aseptically after every 3^rd^ day (Figure S1). In addition, the mycelium growth of *P. ostreatus* and its penetration within the textile mould was further confirmed by SEM (as shown in Figure 2) and FTIR data. Figure 3 demonstrates the FTIR spectra of fungal-grown textiles. In general, the fungal mycelium is composed of fatty acids, amides, and polysaccharides, which corresponds to 3,000–2,800 cm^−1^, 1,700–1,300 cm^−1^ and 1,200–900 cm^−1^, respectively (Tacer-Caba et al. 2020). These are consistent with peaks found in the current study as shown in Figure 3. For instance, a prominent peak at 1,621 and 1,631 cm^−1^ can be observed in coloured and white cotton textiles, respectively.

On the other hand, textiles after the fungal growth (3^rd^ week of incubation) demonstrated a decrease in water activity by 80%–90% compared to control textile (Figure S2). Water activity reflects the amount of water left in the substrate for microorganisms to grow (Agarwal et al. 2020). Hence, a decrease in water activity in samples testifies to the *P. ostreatus* growth in textiles. Moreover, the maximum compressive strength was observed as 270 kPa and 100 kPa in cotton and polyester textiles grown with *P. ostreatus*, respectively (Figure S3). The compressive strength of the textile-based composite can be used as an alternative to polystyrene, as it has been reported to have a range of 69–400 kPa (Amstislavski et al. 2020). In general, the material’s comprehensive strength depends on the type of mycelium-producing strain as well as the substrate. For instance, Tacer-Caba et al. (2020), reported the strength of 300 kPa in rapeseed cake grown with *Trichoderma asperellum*. Similarly, the author reported the 200 kPa of comprehensive strength in rapeseed cake grown with *Agaricus bisporus*. In addition, the availability of carbon sources and supplementation can affect the fungal and mycelium growth rate, thus further affecting the compressive strength of biocomposites. The presence of the high amount of lipids and proteins in mycelium increases the plasticiser properties while polysaccharides provide stiffness to mycelium-based film (Tacer-Caba et al. 2020). Hence, it would be necessary to target the desired product based on material requirements.

In the present study, the textile was supplemented with only 5 g/L of glucose, hence, further supplementing the textile with a sugar-rich substrate such as hydrolysate derived from lignocellulosic biomass will provide the fungus with enough nutrients to grow and produce mycelium to increase lipids and protein concentration ultimately increasing the strength and stiffness of the material, while sustaining the idea of renewable and sustainable-based biocomposite production.

### 3.3. Limitation, scope and future applications

So far, different types of substrates such as rapeseed, oat husk (Tacer-Caba et al. 2020), wheat bran supplemented with cotton powder (Santos et al. 2021), apple residues (Attias et al. 2020), agricultural waste (Pelletier et al. 2017), and footwear waste (Silverman et al. 2020) have been employed to produce mycelium-based biocomposites. However, to the best of our knowledge, research on textile-based biocomposites has not been reported. Additionally, most of the reported studies have been limited to laboratory scale, and the change in mycelium growth pattern upon varying the substrates and/or other additives to vary the biocomposite characteristics has not been investigated (Andrew and Dhakal 2022). Nonetheless, a key result from the present study indicates that *P. ostreatus* can grow in both polyesters and cotton textiles and form biocomposites. Further optimisation would be necessary using process strategies, such as supplementation with other waste materials (e.g. lignocellulosic biomass or food waste), pH or temperature effect to increase the mycelium growth. Material properties can also be optimised by varying the compressive strength, density or flexibility of biocomposite materials based on applications such as food packaging, electrical wires, automobiles and coating. Also, techno-economic analysis and life cycle assessment will provide the economic aspect of biocomposite production and its potential impact on the environment.

## 4. Conclusions

The growth of *P. ostreatus* on textile residue and mycelium production proves the applicability of textile waste as a potential feedstock. However, the loss in biocomposite weight of 1%–5% has been further corroborated by the water loss in the sample and in fungal mycelium. Additionally, the maximum compressive strength was observed as 270 kPa using cotton-based biocomposite. Nonetheless, this short study sets the base for further experiments to develop the textile-based biocomposite with varying properties which will have a great effect on the bioproduction process while providing wide industrial applications.
